# Masks

**Published:** 1876-09

**Authors:** 


### *MASKS.*

If we could only read each other's hearts, we should be kinder to each other. If we knew the woes and bitterness and physical annoyances of our neighbors, we should make allowances for them which we do not know. We go about masked, uttering stereotyped sentiments, hiding our heart-pangs and our headaches as artfully as we can ; and yet we wonder that others do not discover them by intuition. We cover our best feelings from the light ; we do not so conceal our resentments and dislikes, of which we are prone to be proud. Often two people sit close to gether, with "I love you" in either heart, and neither knows it. Either think, "I could be fond ; but what use of wasting fondness on one who does not care for it?" and so they part and go their way alone. Life is a masquerade, at which few unmask, even to their very dearest. And though there is need of much masking, would to heaven we dared show plainly our real faces from birth to death, for then some few, at least would truly love each other.

---

The "Milliner and Dressmaker," published at 131 Mercer Street, New York, at $1.00 a year, is the cheapest and one of the best fashion magazines in this country.

